# A descriptive study of the perceptions and behaviors of waterpipe use by university students in the Western Cape, South Africa

**DOI:** 10.1186/1617-9625-11-4

**Published:** 2013-02-08

**Authors:** Karin E Daniels, Nicolette V Roman

**Affiliations:** 1 Department of Social Work, University of the Western Cape, Modderdam Road, Bellville, Cape Town 7435, South Africa

**Keywords:** Hookah pipe, Water pipe, Tobacco, Health risk

## Abstract

**Background:**

Waterpipe smoking started as a cultural phenomenon but has become a social phenomenon. Hookah cafes are an increasingly popular venue for socializing. Studies suggest that waterpipe users perceive smoking the waterpipe as less addictive and harmful than cigarette smoking. The aim of this study was to assess the beliefs, and associated behaviours, regarding the health-risk of smoking the waterpipe.

**Methods:**

A cross-sectional descriptive study was conducted with a sample of first year students at a historically black university in the Western Cape, South Africa. A self-administered questionnaire was constructed from the College Health Behaviour Survey. The final sample was 389 university students, 64% (250) females and 36% (139) males. The sample had a mean age of 22.2 years (*SD* = 5.04).

**Results:**

Waterpipe users perceived the health risks of smoking the waterpipe to be exaggerated (48%) and less addictive (58%) than non-users (13% and 17%, *p*<.001). Additionally, the findings confirm that waterpipe smoking is conducted in a social setting (61%). This social setting included smoking on campus (28%), in the family home (11%), at a party (9%), at a friend’s place (6%) and in a restaurant (1%). Of concern was the majority of users smoked the waterpipe on a daily basis (70%) and that the tobacco mix was easily available (90%). The most common self-reported reason for smoking the waterpipe was for relaxation.

**Conclusion:**

As with previous studies, the results of this study confirm the false perception that smoking the waterpipe is not a health risk and is socially acceptable. Additionally, the findings of the study raise concerns and an awareness of smoking the waterpipe in the family home and implications for children. The results of this study provide important information for tobacco control and substance abuse policies in South Africa. These findings highlight the need for further research to determine the extent of waterpipe smoking at other universities in South Africa.

## Introduction

The use of the waterpipe (also known as hubbly bubbly, shisha, narghile or hookah pipe) started as a cultural phenomenon [1]. Today the use of the waterpipe has become a social phenomenon as with cigarette smoking, with hookah bars, cafés and restaurants becoming popular social gathering places for young smokers and their friends [2]. One of the reasons for the popularity of the waterpipe is the social availability and accessibility of both the waterpipe and the tobacco used [3]. Furthermore, waterpipe use is widely viewed as a safer alternative to cigarette smoking rather than a potential health risk [4]. Waterpipe smoke contains significantly higher quantities of toxic heavy metals such as arsenic, nickel, cobalt, chromium, lead as compared with cigarette smoke [5]. Research has indicated that the relationship between water pipe use and consumer risk, is dose-response related [6]. The health effects of the waterpipe are under-studied, but users believe that as smoke is drawn through water, the filtration process removes dangerous particles in the smoke, and users would therefore consider waterpipe smoking as a safer alternative to smoking cigarettes [7,8]. However, there are studies which suggest that the level of the nicotine does not change when the smoke is filtered [9,10]. Thus the waterpipe could be considered to be a health risk due to the presence of nicotine and toxic heavy metals in the smoke of the waterpipe. Studies suggest that smoking the waterpipe has long-term health effects which include cancer [11,12], respiratory health issues [13], acute increased heart rate and systolic and diastolic blood pressure [14].

Besides the smoke of the waterpipe being a health risk owing to tobacco use, additional health risks have been noted in studies. Sharing a waterpipe is a contributing factor to the spreading of tuberculosis, mononucleosis, viruses and bacteria when an infected individual shares a mouthpiece with none-infected individuals because of the transmission of oral secretions [15]. The humid closed hose may act as a source of tuberculosis infection among waterpipe users and the common use of one waterpipe amongst a group of users [16]. Poor sanitation, inadequate cleaning of the waterpipe and lack of public health oversight contribute to the spread of infectious diseases. In addition, hookah bars are not required to sterilise or replace the waterpipe mouthpieces after use [15].

Prevalence studies suggest that waterpipe use amongst school children in Middle Eastern countries and among university student groups of Middle Eastern descent in Western countries have the highest rates [2]. In addition, the use of the waterpipe often takes place during social activities between family members and friends, in and out of the home. Smoking the waterpipe predicts regular and increased cigarette smoking [15].

Research focusing on the waterpipe in South Africa is limited. To our knowledge, only two studies focusing on the waterpipe have been conducted in South Africa. The first study focused on secondary school learners in a disadvantaged community in Johannesburg. The results indicated that 60% of participants used the waterpipe, which included 20% daily use [17]. The second study focused on university medical students in Pretoria. The prevalence of waterpipe use was 18.6%. The results suggested that South African medical students used alternative tobacco products and this could be considered to be part of a pattern of risk-taking behaviour [10].

According to the results of the South African Youth Risk Behaviour Survey 2008 conducted by the Medical Research Council [7], the Western Cape Province (36.7%) has a significantly higher prevalence of current tobacco smoking and current frequent tobacco smoking (14.6%) than the national average of 21.0% and 5.8% respectively. Although the South African government has implemented legislative action to discourage tobacco use by increasing taxation and banning advertising, tobacco consumption still remains a public health concern [18,19]. The South African Tobacco Control policy prohibits tobacco smoking in public spaces, but prohibiting waterpipe smoking has not been effected. Although, studies provide sufficient evidence that waterpipe use is a potential health risk, young people in South Africa may not necessarily be aware of the health risks of smoking the waterpipe. The purpose of this study therefore was to determine the risk perceptions and behaviours of university student waterpipe users in the Western Cape in South Africa.

## Methods

This study was a cross-sectional study conducted in the Faculty of Community and Health Sciences with the assumption that students studying in this faculty would be aware of the potential health risks in waterpipe smoking. First year students, from all departments in the faculty, are required to attend the interdisciplinary modules offered in the faculty. As these classes are fairly large in numbers, two of three classes were randomly selected for students to participate in this study. A final sample of 389 of self-selected participants voluntarily participated in this study from a group of 415 students. The final sample included 250 (64%) females and 139 (36%) males with a mean age of 22.2 (*SD* = 5.04) years. Approximately 50% of the sample identified themselves as Coloured (mixed ethnicity) followed by 40% Black African, 6% Whites and 4% Indians.

Data was collected with a self-administered questionnaire. Specific items regarding waterpipe use were taken from the Missouri College Health Behavior Survey (MCHBS, 2010-2011) [20], which was developed at the University of Missouri in Columbia, USA, to construct a questionnaire for this study. Permission to conduct the study was granted by the Institutional Review Board at the University of the Western Cape. A pilot study was conducted with 72 second year students in order to determine if the items in the questionnaire were language appropriate and made sense. The instrument was adapted after the analysis of the pilot study. These changes included the inclusion of additional items concerning the behaviors, perceptions and knowledge of waterpipe smoking. Additionally, the responses to the items were modified from a 3-point to a 5-point likert scale so that it was easier for the participants to understand.

Participants were informed regarding the purpose and ethical considerations of the study, as well as the use of the data for the purpose of publication. Participants therefore provided informed consent to voluntarily participate in the study. The Chi-square (X^2^) tests were used to test for significant differences between users and non-users in terms of perceptions and behavior of smoking the waterpipe. The statistical analysis was conducted with the Statistical Package for Social Science (SPSS v 20.0).

## Results

The results indicated that 40% of the respondents were current waterpipe users. The mean age for first-time waterpipe smoking was 15.7 years. Waterpipe users did not perceive the waterpipe to be a health risk. The perceptions of waterpipe users differed significantly from the perceptions of non-users. Almost 50% of users, as compared to non-users, believed that a waterpipe has less nicotine (43% and 17%, *p*<0.01), the dangers of smoking the waterpipe are exaggerated (48% and 13%, *p*<0.01), and the tobacco toxins are filtered by the water in the waterpipe (44% and 27%, *p*<0.01). The majority of users believed that smoking the waterpipe is not addictive (58% and 20%, *p*<0.01) and that users could quite easily quit (53% and 17%, *p*<0.01). Furthermore, sharing the waterpipe was perceived as not harmful by waterpipe users (34% and 15%, *p*<0.01). More non-users than users believed that long-term health problems such as heart disease (almost 50% and 32.51%, *p*<0.01), lung cancer (57.34% and 39.31%, *p*<0.01) and lung disease (57.33% and 39,36%, *p*<0.01) could develop. The most common reason for smoking the waterpipe was for relaxation (67% and 65%) but this was not a significant finding (Table 1).

**Table 1:** Perceptions of the health risks of the hookah pipe

**Perceptions of hookah pipe use**	**Users n (%)**	**Non-users n (%)**	
	**154 (40%)**	**299 (60%)**	***p *value**
Smoking the hookah pipe helps one to relax	96 (67%)	121 (65%)	0.39
Smoking the hookah pipe helps people stay thin	33 (22%)	38 (17%)	0.13
One gets less nicotine from a hookah pipe	66 (43%)	37 (17%)	<0.01
The hookah pipe is as addictive as cigarettes	48 (31%)	104 (46%)	<0.01
An occasional cigarette is more dangerous than smoking the hookah pipe	56 (36%)	37 (16%)	0.06
The dangers of smoking the hookah pipe are exaggerated	73 (48%)	29 (13%)	<0.01
Sharing the hookah pipe is not harmful to one’s health	53 (34%)	34 (15%)	<0.01
Hookah pipe smokers become more addicted the more they smoke	52 (34%)	122 (53%)	<0.01
Each inhalation of hookah smoking has an effect on the body	51 (33%)	120 (52%)	<0.01
Hookah pipe smoking takes years off a smoker’s life	42 (27%)	65 (28%)	<0.01
Smoking a hookah pipe is not as addictive as smoking cigarettes	90 (58%)	45 (20%)	<0.01
Hookah pipe smokers can quit easily	82 (53%)	39 (17%)	<0.01
Smoke inhaled from the hookah pipe contains harmful chemicals	55 (38%)	111 (49%)	<0.01
Tobacco toxins are filtered out by the water in the hookah pipe	68 (44%)	62 (27%)	<0.01
**Health risks of hookah pipe use**			
The chances that a typical hookah pipe smoker will develop heart disease	32.51%	49.51%	<0.01
The chances that a typical hookah pipe smoker will develop lung cancer	39.31%	57.34%	<0.01
The chances that a typical hookah pipe smoker will develop lung disease	39.36%	57.33%	<0.01

Twenty-eight percent (28%) of waterpipe users smoked the waterpipe on campus. Less than 1% smoked the waterpipe in a restaurant. The majority smoked in a social setting (61%), with other people (82%) and daily (70%). Twenty-one percent of respondents smoked the waterpipe in the family home. Respondents also indicated that the tobacco mix was easily accessible (90%) (Table 2).

**Table 2:** Associated behaviours with waterpipe use

**Behaviours**	**Users**
	**40% (154)**
Places where the waterpipe is smoked^1^	
*At home*	11% (17)
*On campus*	28% (43)
*In a restaurant*	0.7% (1)
*At a party*	9% (13)
*At a friend’s house*	6% (9)
*All of the above*	46% (70)
Settings or occasions when the waterpipe is smoked	
*Family home*	21% (30)
*In a social setting*	61% (88)
*With alcohol consumption*	13% (18)
*After meals*	5% (7)
Frequency of waterpipe smoking	
*Daily*	70% (108)
*Once a week*	17% (26)
*Every 2 weeks*	3% (4)
*Once a month*	2% (3)
Waterpipe used with others	82% (124)
Waterpipe used alone	18% (27)
Tobacco mix easily available	90% (140)

^1^ The section for “Places where the waterpipe is smoked” contains 153/154 responses for these items on the questionnaire. The next sections are different items within the questionnaire and therefore do not equate to 154.

## Discussion

This study describes the perceptions and behaviours of waterpipe smoking (also known as shisha, hubbly bubbly, narghile or hookah pipe) by university students in the Western Cape. The percentage of waterpipe smokers (40%) in our study sample was higher than the 18.6% prevalence in the study by Senkubuge, et al. [10] but lower than the 60% prevalence in the Combrink et al. [17] study, both conducted in the northern provinces of South Africa. The age of onset for waterpipe smoking in our study was 15.7 years, which is comparable with other studies^15^.

Overall, students did not perceive waterpipe smoking to be a health risk since most of them believed the dangers of smoking the waterpipe to be *exaggerated*. In addition, students believed that there were no long-term addictive and health effects from using the waterpipe. As with studies conducted locally and internationally, students could therefore perceive the waterpipe as less harmful than cigarette smoking [7,10,11,17] possibly owing to the belief that water in the waterpipe filters out the tobacco toxins [9]. However, previous studies have suggested that waterpipe and cigarette smoking share similar health risks, with more carbon monoxide, similar nicotine and more smoke exposure in waterpipe smoking [5,9]. A recent study further suggests that early onset of cancer is indicated in oral lesions possibly due to waterpipe use [12].

Given the potential health risks of waterpipe smoking, the behavioural patterns found in our study raise concerns. The majority of waterpipe smoking occurred alarmingly on a daily basis, which is much higher than the findings of other studies [8]. Our study also confirmed the social acceptability of waterpipe smoking and easy accessibility of the tobacco used in the waterpipe, as found in previous research [2,3]. Since hookah bars, cafés and restaurants are considered the most prevalent social places for waterpipe smoking, our study unexpectedly highlights the high rates of use on campus, rather than in restaurants. This high rate could be due to the social acceptability and non-prohibition of tobacco policies on the use of the waterpipe [8]. In other words, since the waterpipe is not mentioned in any of the South African policies of tobacco control or substance abuse, there seems an acceptability of public use of the waterpipe. The South African Tobacco Control policy [19] prohibits tobacco smoking in public spaces, but prohibiting waterpipe smoking has not been effected or considered in the policies. This non-prohibition could add to the possible misperception that smoking the waterpipe is not a health risk, and is therefore an acceptable social phenomenon. Of serious concern, is the permissibility of waterpipe smoking in the family home. Even though the majority did not smoke the waterpipe in the family home, this finding also raises concerns in terms of early onset of waterpipe use amongst children and youth especially since a study in South Africa raises similar concerns [10,17]. Family members could be users and therefore initiators and supporters of early onset of waterpipe use among youth [8].

Study limitations include the target sample of the study and the cross-sectional design of the study. This study was conducted with students registered with one faculty at one university. Perhaps a broader target sample could affect the prevalence rates of waterpipe users attending universities as there may be differing university tobacco policies, which could constrain waterpipe users. This was a cross-sectional study and therefore only provides a snapshot of an occurrence. However, this study is the start to an explorative process to understand the extent of the problem of waterpipe users. Additionally, this study provides key findings in terms of the perceptions and behaviours regarding waterpipe smoking by university students in South Africa.

## Conclusion and recommendations

The results of this study confirm the false perception that waterpipe smoking is harmless and non-addictive when compared to cigarette smoking, and in addition highlights the alarmingly high rates of daily use on campus. These findings highlight the need for further research to determine the extent of waterpipe smoking at other universities and within the public arena in South Africa. A review of the South African tobacco policies should include and regulate water pipe smoking. Specifically, restraints should be imposed on hookah bars, cafés and restaurants in the same way as tobacco control policies for cigarette smoking. A revision of university policies is needed in order to incorporate waterpipe smoking within the policies and thus constrain the public usage thereof. Furthermore, health education programmes regarding this potentially harmful activity need to be all-encompassing about the dangers of waterpipe smoking, and should be implemented with much younger youth, especially since the age of onset is earlier than 18 years.

## Competing interest

We do not have competing interest for this study.

## Authors’ contributions

Author, KD, conducted the study and constructed the article. Author, NR, analysed, interpreted and revised the article. Both authors read and approved the article for submission.
